# Bacterial colonisation of surface and core of palatine tonsils among Tanzanian children with recurrent chronic tonsillitis and obstructive sleep apnoea who underwent (adeno)tonsillectomy

**DOI:** 10.1017/S0022215123001147

**Published:** 2024-01

**Authors:** Denis R Katundu, Desderius Chussi, Christa E van der Gaast-de Jongh, Maroeska M Rovers, Marien I de Jonge, Gerjon Hannink, Niels van Heerbeek

**Affiliations:** 1 Department of Otolaryngology, Kilimanjaro Christian Medical Centre, Kilimanjaro, Tanzania; 2 Department of Otolaryngology, Kilimanjaro Christian Medical University College, Kilimanjaro, Tanzania; 3 Department of Otolaryngology, Head and Neck Surgery, Radboud University Medical Center, Nijmegen, the Netherlands; 4 Department of Laboratory Medicine, Laboratory of Immunology, Radboud Centre for Infectious Diseases, Radboud Institute for Molecular Life Sciences, Radboud University Medical Center, Nijmegen, the Netherlands; 5 Department of Medical Imaging, Radboud Institute for Health Sciences, Radboud University Medical Center, Nijmegen, the Netherlands

**Keywords:** Bacterial, surface, core, tonsil, children, adenotonsillectomy, Africa

## Abstract

**Objective:**

Acute and chronic tonsillitis are frequently treated with antibiotics. This study aimed to understand the presence of pathogenic micro-organisms on the surface and core of chronically infected tonsils among Tanzanian children.

**Methods:**

The study enrolled children undergoing adenotonsillectomy. Surface and core tonsillar swabs were taken. Quantitative polymerase chain reaction was performed for *Streptococcus pneumoniae*, *Haemophilus influenzae*, *Moraxella catarrhalis*, *Staphylococcus aureus*, *Neisseria meningitidis* and *Pseudomonas aeruginosa*.

**Results:**

Surface and core combined, isolated *N meningitidis* (86.1 per cent) was found the most, followed by *H influenzae* (74.9 per cent), *S pneumoniae* (42.6 per cent) and *S aureus* (28.7 per cent). *M catarrhalis* and *P aeruginosa* were only found in a few patients, 5.6 per cent and 0.8 per cent respectively.

**Conclusion:**

Colonisation of the tonsillar surface and core has been found. Potentially pathogenic micro-organisms are likely to be missed based on a throat swab. Hence, the practice of surface tonsillar swabbing may be misleading or insufficient.

## Introduction

The palatine tonsils are lymphoid tissues, which are components of the mucosa-associated lymphoid tissue of the pharynx, and part of Waldeyer's tonsillar ring, being responsible for immune surveillance of the upper respiratory tract.^1^ Despite being part of the first line of defence, the tonsils frequently get infected. Acute and chronic tonsillitis have been one of the most common reasons for children to seek otorhinolaryngological attention globally.

Both acute and chronic tonsillitis are frequently treated with antibiotics.^2–4^ Antibiotics used to treat tonsillitis will not only target micro-organisms on the surface of the tonsils but also those in the tonsillar crypts (or core), leading to complete sterilisation.^5^ However, for centuries there has been discussion on the presence and identity of micro-organisms on the surface and in the core of the tonsils, and their role in causing tonsillitis, as studied in different settings.^6–8^ It therefore is important to understand the presence and identity of pathogenic micro-organisms on the surface and in the core of extracted tonsils in Tanzanian children in order to understand different attitudes and practices regarding antibiotics prescription and intake. Paediatric tonsillectomy, being the major oropharyngeal surgery conducted in Tanzanian consultant hospitals, offers the ideal setting to answer this question.^2,9–12^

It would also be valuable to determine the rationale of antibiotic treatment in tonsillitis, as evidence for the effectiveness of antibiotics is scarce or even lacking in most cases of acute or chronic tonsillitis. The risks of needless antibiotic exposure and the contributing likelihood of antimicrobial resistance should not be underestimated.^13–15^

This study aimed to understand bacterial colonisation of the surface and the core of palatine tonsils in a paediatric population in Tanzania, in patients with chronic recurrent tonsillitis and/or obstructive sleep apnoea syndrome as a result of tonsillar hypertrophy scheduled for adenotonsillectomy.

## Materials and methods

As part of a non-inferiority, double-blind, placebo-controlled, randomised, controlled trial studying the effect of post-operative antibiotics in children, aged 2–14 years, who were undergoing adenotonsillectomy for sleep apnoea and/or recurrent or chronic tonsillitis, both tonsil surface and core swabs were obtained. Patient inclusion was as per the published study protocol.^16,17^

All swabbing was carried out under general anaesthesia (by author DRK). Adenotonsillectomy was performed by different surgeons. Following induction of anaesthesia and orotracheal intubation, the patient was placed in the tonsillectomy position with the oropharynx exposed using a mouth gag. Then, using a sterilised cotton-tipped applicator, surface tonsillar swabs were taken on both sides with the same applicator. Subsequently, tonsillectomy was performed using a technique of the surgeon's experience and preference. The dissected tonsils were dipped in povidone for about 30 seconds and rinsed out using sterilised physiological normal saline. Afterwards, using a sterile surgical blade, both tonsils were divided into two parts, and the samples were taken from the core of the tonsils again with the same single applicator. Both the surface and the core swabs were immediately immersed, separately, in Copan Universal Transport Medium (Brescia, Italy) for preservation and transport. All swabs were immediately stored at −80 degrees.

Quantitative polymerase chain reaction for *Streptococcus pneumoniae*, *Haemophilus influenzae*, *Moraxella catarrhali*s, *Staphylococcus aureus*, *Neisseria meningitidis* and *Pseudomonas aeruginosa* was performed on all swabs, as previously described.^18^ Stored swab samples were thawed on ice and vortexed.^18^ From each sample, 100 μl was aliquoted into a 96-well plate. The plate was incubated for 15 minutes at 93°C, to lyse the bacteria. The quantitative polymerase chain reaction was performed in six monoplex reactions, using the Bio-Rad CFX96 Touch^™^ real-time quantitative polymerase chain detection system. All reactions were performed in a 10 μl final volume containing 1 μl bacterial lysate, 5 μl SsoAdvanced^™^ Universal Probes Supermix (Bio-Rad), 400 nM of each primer and 200 nM probe (see supplementary material for primers and probe details). Every 96-well plate contained, in duplicate, a no-template control, and a seven-step, 10-fold serial dilution of a positive control, starting at approximately 10 ng purified DNA. The quantitative polymerase chain reaction programme consisted of 3 minutes of incubation at 95°C, followed by 50 cycles of 10 seconds at 95°C and 20 seconds at 60°C. Fluorescence was measured after each cycle. In order to accurately compare results within targets, the baseline threshold was adjusted so that the positive controls had the same quantification cycle (Cq) value per target for all plates. The Cq cutoff was set at 36 for each target.

### Statistical analysis

Descriptive statistics were used to summarise the data. McNemar's tests were used to compare differences between tonsillar surface and tonsillar core pathogen counts (i.e. positive polymerase chain reaction results). Subgroup analyses were performed based on surgical indication (i.e. recurrent tonsillitis, paediatric obstructive sleep apnoea or both). Statistical analyses were performed using R software (version 4.2.0; R Foundation for Statistical Computing, Vienna, Austria). A *p*-value of less than 0.05 was considered statistically significant.

## Results

Both surface and core swabs were obtained from 251 tonsil pairs. Patient characteristics are shown in Table 1. Among the participants, 8 per cent were not yet enrolled in school. Gender distribution was almost equal, as was urban–rural distribution. As expected, no grade 1 tonsils (i.e. within the normal range) were seen in clinic or during surgery. Nearly 98 per cent of participating children had not previously been exposed to ENT surgery. Most children had both indications for surgery; that is, recurrent tonsillitis as well as clinical signs and symptoms of paediatric obstructive sleep apnoea (45.8 per cent). The majority of surgeons preferred electrodissection as the modality of tonsillectomy (Table 1).

**Table 1.** Participants' characteristics

Characteristics	Values
Age at surgery (mean (SD); years)	4.86 (2.45)
Sex (*n* (%))	
– Female	117 (46.6)
– Male	134 (53.4)
Body weight (mean (SD); kg)	18.94 (6.47)
Education (*n* (%))	
– Not enrolled in school	20 (8.0)
– Primary	105 (41.8)
– Pre-school	126 (50.2)
Residency (*n* (%))	
– Rural	122 (48.6)
– Urban	129 (52.4)
Indication (*n* (%))	
– Recurrent chronic tonsillitis	45 (17.9)
– OSA	91 (36.3)
– Both	115 (45.8)
Brodsky tonsil grading score (*n* (%))	
– Grade 2	7 (2.8)
– Grade 3	120 (47.8)
– Grade 4	124 (49.4)
Previous ENT surgery (*n* (%))	6 (2.4)
Surgical modality (*n* (%))	
– Classical dissection	29 (11.6)
– Guillotine	11 (4.4)
– Electrodissection	209 (83.3)
– Combined	2 (0.8)
Study centre (*n* (%))	
– Arusha Lutheran Medical Centre	42 (16.7)
– Kilimanjaro Christian Medical Centre	209 (83.3)

SD = standard deviation; OSA = obstructive sleep apnoea

Regarding the total number of pathogens, surface and core combined, isolated *N meningitidis* (86.1 per cent) was found the most, followed by *H influenzae* (74.9 per cent), *S pneumoniae* (42.6 per cent) and *S aureus* (28.7 per cent), as shown in Table 2. *M catarrhalis* and *P aeruginosa* were only found in a few patients, 5.6 per cent and 0.8 per cent respectively. Comparing the different locations (surface *vs* core), significantly more pathogens were isolated from the surface than from the core. Regarding the different pathogens, we found *N meningitidis* in both swabs or on the surface only, but hardly ever in the core only. *H influenzae* was found mainly in both swabs, less frequently in the core alone and even less frequently on the surface alone. *S pneumoniae* was found most frequently on the surface only, less in both swabs and hardly ever in the core only. *S aureus* was mainly found in the core and less often in the surface or in both swabs. *M catarrhalis* was almost only found on the surface, and the only two cases with *P aeruginosa* were found in the core.

**Table 2.** Tonsil pathogens isolated from surface, core or both

Bacterial species	Surface only	Core only	Both	Total	*P*-value
*Haemophilus influenzae*	14 (5.6)	35 (13.9)	139 (55.4)	188 (74.9)	0.004
*Moraxella catarrhalis*	12 (4.8)	1 (0.4)	1 (0.4)	14 (5.6)	0.005
*Neisseria meningitidis*	106 (42.2)	6 (2.4)	104 (41.4)	216 (86.1)	<0.005
*Streptococcus pneumoniae*	66 (26.3)	6 (2.4)	35 (13.9)	107 (42.6)	<0.005
*Pseudomonas aeruginosa*	0 (0.00)	2 (0.8)	0 (0.0)	2 (0.8)	0.47
*Staphylococcus aureus*	18 (7.2)	39 (15.5)	15 (5.98)	72 (28.7)	0.008
Total (*n*)	216	89	294	599	

Data indicate numbers and percentages, unless indicated otherwise.

Co-existence of the studied pathogens has been noticed. In the surface swabs, no pathogens were detected in 8.4 per cent of the patients. In the core swabs, no pathogens were found in 15.9 per cent of the patients. Two or more pathogens were found more often in the surface swabs as compared to the core swabs (70.5 per cent *vs* 48.2 per cent, respectively). The co-existence of *H influenzae* with *N meningitidis* (21.5 per cent), and of *H influenzae* with *N meningitidis* and with *S pneumoniae* (20.7 per cent) was found most in the surface swabs. The co-existence of *H influenzae* with *N meningitidis* (17.1 per cent), and of *H influenzae* with *N meningitidis* and with *S aureus* (7.2 per cent) was observed most often in the core swabs. All other combinations can be found in the supplementary material. No differences in colonisation were found between the different surgical indications.

## Discussion

Tonsillectomy due to chronic recurrent tonsillitis remains one of the most common surgical procedures performed in childhood worldwide. Findings from this study add value to the understanding of differences in bacterial colonisation of the surface and the core of the palatine tonsils of children from resource-limited settings.^17,19–21^

This study focuses on the most common potentially pathogenic bacteria. In the upper respiratory tract, *N meningitidis*, *H influenzae* and *S pneumoniae* were observed to be the most common species in both tonsil sites. More species were identified in the surface than in the core swabs. Although this can be explained by the differences in size of the swab area, it is more likely that the surface just harbours more micro-organisms than the core. Another typical finding was that the distribution of species was significantly different for the two sites. A predominance of *S aureus* and *H influenzae* in the core was found, while *N meningitidis* was predominantly found on the surface. These findings prove the uniqueness of both swab types, indicating that a microbiology throat swab alone, in children with acute bacterial infection on chronically infected tonsils, might not provide sufficient treatment standpoints and can hence be misleading. The same is true for performing a core swab only. Findings from the current study are in accordance with other recently published studies, including those conducted in low to middle income countries.^4,6,20,22–25^ However, some studies could not find differences in microbial composition between surface and core swabs. This can be explained by their small sample sizes and/or the employed laboratory techniques.^26,27^

Many of the studied swabs contained between two and four different bacterial species. Limited studies have been conducted on this polymicrobial co-existence. This detection was possible because of the employed polymerase chain reaction technique, which is superior over standard culture testing, where the presence of different pathogens can be masked because of the overgrowth of one specific pathogen.^23^ Identified polymicrobial potential pathogens may play a role in the aetiopathogenesis of serious acute tonsil infections, which may complicate, for example, peritonsillar, retropharyngeal, neck, lung and brain abscesses. Polymicrobial co-existence may contribute to increased tonsil sizes and emerging bacterial biofilms among children with chronic recurrent tonsilitis.^28–33^

Children with clinical features of acute bacterial tonsillitis or acute complication(s) of chronic recurrent tonsillitis may have negative surface swab findings but still harbour pathogenic organism(s) in the tonsil core. Obtaining core swabs is not practical, and, as swab microbiology has a turnaround time of 48–72 hours, clinicians should be cautious when initiating antibiotics. Clinicians can target micro-organisms isolated from this study while waiting for swab results.

The current study has the advantages of a large sample size and participant homogeneity. The co-existence of different bacterial species highlights the possibilities of synergisms and polymicrobial colonisation, and might help in choosing the most appropriate antimicrobial treatment regimen. Further studies shall be designed to investigate the virulence of these micro-organisms in children in the two sites (tonsil surface and core), as well as examining the role of viruses and fungi in paediatric (adeno)tonsillitis. Most (adeno)tonsillitis cases are assumed to be of viral origin. Future studies may look for associations of bacterial co-existence and their effects on palatine tonsil mass. Findings from this study shall be translated with caution for generalisability. This is because the microenvironment of the tonsil surface might attract different pathogens than the microenvironment of the tonsil core, but the opposite might also be true. Hence, there may (or will) be differences in the type (and number) of pathogens found throughout the world between the two tonsil sites. Some pathogens are simply found more in certain parts of the world than others.

- Both acute and chronic tonsillitis are frequently treated with antibiotics
- Antibiotics not only target micro-organisms on the surface of the tonsils but also those in tonsillar crypts
- Understanding pathogenic micro-organisms for chronic tonsillitis in children from low to middle income countries is important
- Colonisation of the tonsillar surface and core has been found
- Potentially pathogenic micro-organisms are likely to be missed based on throat swabbing
- The practice of surface tonsillar swabbing may be misleading or insufficient

## Conclusion

Various patterns of colonisation of the tonsillar surface and core by bacterial species have been found in this study. Potentially pathogenic micro-organisms are likely to be missed based on throat swab analysis only, as bacterial infection may result from the species residing in the crypts of the tonsils rather from those on the surface. Hence, the result of a surface tonsillar swab may be misleading or insufficient. In light of the observed polymicrobial co-existence and the differences in surface and core swab results, we suggest that, in cases of aggressive tonsillitis, imminent complications or suspected antimicrobial unresponsiveness, the identified bacterial species from this study be considered in the aetiology, instead of relying solely on surface swab culture results.

## Supporting information

Katundu et al. supplementary material Katundu et al. supplementary material

## Supplementary material

The supplementary material for this article can be found at https://doi.org/10.1017/S0022215123001147
