# Supplementary material for: Bacterial colonisation of surface and core of palatine tonsils among Tanzanian children with recurrent chronic tonsillitis and obstructive sleep apnoea who underwent (adeno)tonsillectomy
Source: J Laryngol Otol. 2023 Jun 19;138(1):89–92. doi: 10.1017/S0022215123001147 (PMC10772025; doi:10.1017/S0022215123001147)
Supplement: Katundu et al. supplementary material [file S0022215123001147sup001.docx]

Supplementary: Distribution and co-existence of pathogens between the surface and core of tonsils

| **Co-existence of microorganism** | **N**  **species** | **Surface swab**  **N (%)** | **Core swab**  **N (%)** |
| --- | --- | --- | --- |
| None | 0 | 21 (8.4) | 40 (15.9) |
| *S. aureus* | 1 | 2 (0.8) | 8 (3.2) |
| *H. influenzae* | 1 | 11 (4.4) | 63 (25.1) |
| *N. meningitidis* | 1 | 38 (15.1) | 15 (6.0) |
| *S. pneumoniae* | 1 | 2 (0.8) | 4 (1.6) |
| *H. influenzae* & *N. meningitidis* | 2 | 54 (21.5) | 43 (17.1) |
| *H. influenzae* & *S. aureus* | 2 | 0 (0) | 13 (5.2) |
| *H. influenzae* & *S. pneumoniae* | 2 | 3 (1.2) | 10 (4.0) |
| *N. meningitidis* & *S. aureus* | 2 | 6 (2.4) | 8 (3.2) |
| *N. meningitidis* & *M. catarrhalis* | 2 | 1 (0.4) | 0 (0) |
| *N. meningitidis* & *S. pneumoniae* | 2 | 25 (10) | 2 (0.8) |
| *H. influenzae* & *M. catarrhalis* & *N. meningitidis* | 3 | 6 (2.4) | 1 (0.4) |
| *H. influenzae* & *M. catarrhalis* & *S. pneumoniae* | 3 | 1 (0.4) | 1 (0.4) |
| *H. influenzae* & *N. meningitidis* & *S. aureus* | 3 | 11 (4.4) | 18 (7.2) |
|  |  |  | 1 (0.4) |
| *H. influenzae* & *N. meningitidis* & *S. pneumoniae* | 3 | 52 (20.7) | 16 (6.4) |
| *H. influenzae* & *S. pneumoniae* & *S. aureus* | 3 | 1 (0.4) | 1 (0.4) |
| *N. meningitidis* & *S. pneumoniae* & *S. aureus* | 3 | 2 (0.8) | 0 (0) |
| *H. influenzae* & *S. pneumoniae* & *P. aeruginosa* | 3 | 0 (0) | 1 (0.4) |
| *M. catarrhalis* & *N. meningitidis* & *S. pneumoniae* & *S. aureus* | 4 | 1 (0.4) | 0 (0) |
| *H. influenzae* & *M. catarrhalis* & *N. meningitidis* & *S. pneumoniae* | 4 | 4 (1.6) | 0 (0) |
| *H. influenzae* & *N. meningitidis* & *S. pneumoniae* & *S. aureus* | 4 | 10 (4.0) | 6 (2.4) |
